# Adolescent-Reported Food Insecurity: Correlates of Dietary Intake and School Lunch Behavior

**DOI:** 10.3390/ijerph18126647

**Published:** 2021-06-21

**Authors:** Naomi Nichele Duke

**Affiliations:** 1 Division of General Pediatrics and Adolescent Health, Duke Center for Childhood Obesity Research, Department of Pediatrics, Duke University School of Medicine, Box 3675 Duke University Medical Center, Durham, NC 27710, USA; naomi.duke@duke.edu; 2 Department of Sociology, Duke Trinity College of Arts and Sciences, Durham, NC 27708, USA

**Keywords:** adolescent, dietary intake, food insecurity, free and reduced-price lunch, minnesota student survey, nutrition, diet-related health risks

## Abstract

Much of what is known about food insecurity (FI) experiences for young people is based on caregiver report. As such, our understanding of relationships between youth FI and dietary intake (DI) may be limited, particularly among adolescents who often eat away from home. This study examined relationships between youth-reported past-month FI, past-week DI, and school lunch behavior. Data are from middle and high school participants in the 2019 Minnesota Student Survey (*N* = 125,375), one of the longest-running youth surveys in the US. Logistic regression assessed relationships between FI and DI, including fruit, vegetable, milk, sugar-sweetened beverage (SSB), and fast food consumption, and school lunch behavior, adjusting for demographic, physical, and emotional health indicators. Past-month FI was associated with reduced odds of meeting minimum thresholds for daily fruit, vegetable, and milk intake, and increased odds of daily SSB and frequent fast food consumption. Among food-insecure students, no participation in the National School Lunch Program (NSLP) or NSLP participation uncertainty was associated with increased odds of skipping lunch. Findings suggest the importance of clinical and community innovations to prevent the loss of nutritional quality in favor of energy density for youth and families experiencing FI.

## 1. Introduction

Disruptions in access to diverse and nutrient-rich foods may have long-lasting impact on health [1,2]. For adolescents, a population with increasing autonomy of food and beverage intake, food insecurity (FI) experiences may negatively impact diet-related behaviors during a time when dietary consumption is particularly relevant for future adult health. Much of what we know about FI prevalence and diet-related behaviors is based on adult report, including parents and caregivers as heads of households; however, for older children, FI reports between adults and youth may be incongruent [3]. We know less about relationships between family FI and food-related behaviors among adolescents from their perspective.

Among adults, there is evidence for an association between FI and low-quality dietary intake, including lower intake of beneficial fruits, vegetables, and dairy [4,5,6,7,8], and higher sugar-sweetened beverage (SSB) consumption [8]. Spees and colleagues note that compared to food-secure households, the sources of food for marginal and very low food-secure families more often include convenience stores and fast food restaurants [9]. Indeed, according to recent findings from the National Household Food Acquisition and Purchase Survey, compared to food-secure households, adults in food-insecure households spend more of the budget at convenience stores and household members have more limited availability of fruit and protein as part of their total caloric intake [10]. Despite their accessibility, convenience stores are less likely to offer fresh produce, lean meats and whole grains [11].

Disparities in the quality and quantity of foods between food-secure and food-insecure households are known; however, evidence for a direct link between FI and differences in dietary intake among youth in food-secure versus food-insecure households is more limited [4,5,12]. Current research is perhaps most compelling for an adverse relationship between FI and youth fruit [4,13] and vegetable intake [13,14]. Among school-age children and early adolescents, Lee and colleagues note that during the summer months, youth from food-insecure households consume fewer servings of whole fruits and more SSB on weekends (but not weekdays) compared to youth from food-secure households [15]. Lower availability of healthy foods in food-insecure households is associated with youth lower intake of fruit and vegetable servings [16]. A study of FI and dietary quality among Mexican children and adolescents extends the negative associations between FI and fruit and vegetable intake, to include some protein sources, and for older children and adolescents, dairy intake [17].

As noted above, historically, the leading research addressing FI prevalence and links between FI and youth health and diet-related outcomes prioritizes adult and caregiver account of household FI [18,19]; as such, the literature would benefit from additional perspectives [20]. Research suggests that youth are aware of household FI and are able to report on individual and household FI experiences [14,21,22]. Further, there may be disagreement between youth and parent report [23], such that our understanding of youth FI experiences and diet-related behaviors is incomplete. This may be particularly relevant for adolescents, who often consume food and beverage outside of the home. Further, the proximity of adolescence to adulthood may portend risk associated with the translation of poor dietary outcomes into later disparities in cardiometabolic health [13]. Finally, the literature examining relationships between FI and diet quality and quantity frequently incorporates time frames that may not overlap (e.g., any food insecurity—past 12 months, and dietary recall—last 24 h) [4]. The present study seeks to add to the literature by focusing on adolescent reports, and examining relationships between family FI and individual diet-related behaviors that are concurrent in time.

Using data from a statewide survey of middle and high school students, the current analyses address the following questions: (1) What is the relationship between youth-reported past-month FI and past-week food and beverage consumption, including meeting minimum thresholds for fruit and vegetable consumption, any milk intake (as a proxy for dairy), and intake of SSB and fast food? (2) Are relationships between youth FI and consumption robust to the addition of other hypothesized correlates of food intake, including body mass index (BMI), physical activity, sleep (related to intake timing and food option availability), and mood (a marker of stress and emotion)? Additional analyses examined relationships between FI and student report of school lunch behavior. Examining relationships between FI and food and beverage consumption among adolescents using self-report may offer insights for tailoring anticipatory guidance strategies and facilitating advocacy of food policy systems that assist youth and families in improving dietary access without sacrifice of nutrient density in contexts of resource limitation. It was hypothesized that youth report of past-month FI would be significantly associated with poor diet-related behaviors among adolescents, and that these relationships would be robust to physical and emotional health indicators.

## 2. Materials and Methods

### 2.1. Study Design and Population

This study is a secondary analysis of data from the 2019 Minnesota Student Survey (MSS). The MSS is a population-based, triennial survey administered to students attending public, tribal, and charter schools in Minnesota. It is one of the longest-running youth surveys in the United States, administered since 1989. The MSS represents a collaboration between the Departments of Education, Health, Human Services, and Public Safety. Since its beginning, data from the MSS have been instrumental in following trends in youth behaviors and identifying programming to address youth needs at school, community, and state agency levels [24]. Surveys query students’ positive and adverse experiences in the home, school, and community domains, health- and risk-related behaviors, and perceptions of self-efficacy. The breadth and depth of content available through the MSS establishes its salience in understanding correlates of youth development, health, and well-being.

The MSS is administered to youth at elementary, middle and high school levels. Prior to each administration, all school districts are invited to participate. Participation is voluntary. For participating districts and schools in 2019, the MSS was administered online to 5th-, 8th-, 9th-, and 11th-grade students via passive parental consent (prior to survey administration, parents may view survey content and opt their child out if desired) and student assent. In 2019, 64% of total student enrollment participated in the MSS (81% of public school districts), including 66% of all 5th graders, 68% of all 8th graders, 66% of all 9th graders, and 54% of all 11th graders. Information on parents and students opting out of participation is not collected. Surveys are anonymous. Students taking the survey are advised of their right to skip any question they do not want to answer, and the ability to stop participation in the survey at any time. With each survey administration, MSS data analysts examine surveys for patterns of missingness and patterns of improbable and/or inconsistent answers. Prior to release of the 2019 data, approximately 4% of surveys were removed to preserve data fidelity [25]. The current study uses data from students in the 8th, 9th, and 11th grades, *N* = 125,375. Depending on the school district in Minnesota (as in school districts across the United States), 8th grade may represent the last year of middle school or the second year of junior high school (grades 7–9). Ninth grade may represent the last year of junior high school or the first year of high school. As such, the MSS inclusion of 8th, 9th, and 11th grades provides broad representation for students across middle, junior, and high school levels. The age range, 12–19 years, is also representative of the adolescent age range for secondary schools (grades 7–12). Additional information about the MSS design, administration, data management, and data access is published elsewhere [26,27].

The Minnesota Department of Education provides oversight and approval for all activities related to administration of the MSS. All analyses for the current study were performed according to established ethical guidelines. MSS data were secured with the assistance of the Minnesota Department of Health, Center for Health Statistics, after submission of a research data request form and completion of a data use agreement. The current study was approved by the Duke University Institutional Review Board (Protocol Approval: 2020-0459).

### 2.2. Measures

Students responded ‘yes’ or ‘no’ to the following question about food insecurity (FI): ‘During the past 30 days, have you had to skip meals because your family did not have enough money to buy food?’ This single item corresponds to an item contained in a validated 9-item measure of food insecurity for children ages 12 and older to represent FI (note: response options for the validated survey are ‘never’, ‘sometimes’, and ‘a lot’) [28,29]. Based on the Radimer/Cornell Hunger Scale [30], the MSS FI question conveys severe FI, indicating household food disruption resulting in youth reductions in quantity of food consumption.

Dietary outcomes included fruit, vegetable, milk, sugar-sweetened beverage (SSB), and fast food consumption. Food and beverage intake were measured with the following stem: ‘During the past 7 days, how many times did you….’ For fruit, students were asked ‘eat fruit (do not count fruit juice).’ For vegetables, students were asked ‘eat green salad, potatoes, carrots or other vegetables (do not count French fries, fried potatoes, or potato chips)’. For milk, students were asked ‘drink a glass of milk (count the milk you drank in a cup, from a carton, or with cereal)’. Sugar-sweetened beverages included pop or soda (not counting diet); sports drinks (not counting low calorie); energy drinks; coffee or tea (not counting drinks with artificial sweeteners or diet drinks); and sweetened fruit drinks (not counting 100% fruit juice). For fast food, students reported intake from a fast food restaurant, including carryout or delivery. Response options for all intake included ‘I did not eat or drink this’, ‘1 to 3 times in the last 7 days’, ‘4 to 6 times in the last 7 days’, ‘1 time per day’, ‘2 times per day’, ‘3 times per day’, and ‘4 or more times per day’. Frequencies for intake at all levels for response options were examined, given the natural order (low to high) for the intake outcomes. Based on intake frequencies, final outcome variables were dichotomized to represent minimal recommended intake for fruits (at least two servings/day versus less than this amount) [31] and vegetables (at least three servings/day versus less than this amount [31], any intake of milk (representing the single dairy option in the survey), and one or more servings of SSB/day versus less than this amount [32]. This SSB metric far exceeds the joint recommendation from the American Academy of Pediatrics and the American Heart Association of consuming no more than 8 ounces of sugary drinks per week [33]. Fast food consumption was dichotomized to represent evidence suggesting a pattern of behavior, consumption of fast food four or more times in the past week versus less than this frequency.

The MSS does not include a specific measure for eating breakfast, dinner or family meals. The survey does include a question asking students where they usually get their lunch during a typical school week. For the current analysis, an additional outcome indicating whether or not a student usually ate lunch was included (i.e., students indicating ‘they usually did not eat lunch’ versus choosing other options of where foods are obtained, e.g., school cafeteria, a la carte line, school store or vending machine, somewhere outside of school, and/or bring lunch from home).

Multivariable analyses included sociodemographic covariates: age (continuous variable); self-identified biological sex; self-identified race-ethnic group (Hispanic, Latino/a; Non-Hispanic [NH] American Indian or Alaska Native; NH Asian or Asian American; NH Black, African or African American; NH Native Hawaiian or other Pacific Islander; NH multiple races; and NH White [comparison group due to size and group with lowest reported food insecurity]); free or reduced-price lunch (yes; don’t know; no [comparison group]); and region (Twin Cities metropolitan area versus all other areas of the state, a demarcation set by data administrators). Given the potential for variation in marketing exposure, preferences and availability of foods and beverages, final analytic models included all 8th-, 9th-, and 11th-grade students with adjustments for age, biological sex, race-ethnic identification, receipt of free or reduced-price lunch (the socioeconomic indicator in the data), and region of residence. An additional variable related to housing stability was included in analyses, posited to have relevance for students’ access to food-stable environments. Students reported whether in the past 12 months they had ‘stayed in a shelter, somewhere not intended as a place to live, or someone else’s home because [they] had no other place to stay’. The variable was dichotomized to reflect any instability in housing (i.e., any yes answer) versus no instability in housing over the past year.

Multivariable analyses included additional covariates hypothesized to be related to food and beverage consumption among adolescents. Students were asked to add up all of the time spent in any kind of physical activity that increased their heart rate and made them breathe hard (activity lasting for at least 60 min). Physical activity was included as a dichotomous variable representing activity for 5–7 days versus 0–4 days in the past week. Students indicated the number of hours of sleep during a typical school night: ‘4 or less hours’, ‘5 hours’, ‘6 hours’, ‘7 hours’, ‘8 hours’, ‘9 hours’, and ‘10 or more hours’. Sleep was dichotomized to indicate meeting a minimum threshold for recommended sleep duration for adolescents ‘8 or more hours’ versus ‘less than 8 hours’ in a typical school night [34,35]. Students were asked about feelings of being down, depressed or hopeless and having little interest in doing things over the past two weeks, corresponding to components of the Patient Health Questionnaire-2 (PHQ-2) which has been validated in adolescents [36]. A variable indicating screening positive on the PHQ-2 was created to represent a proxy for low mood-emotional distress. MSS data administrators included a categorical measure for student BMI (calculated from student-reported height and weight) in the data release. In multivariable analyses, students categorized as normal/underweight (single category in data, BMI < 85%) were the comparison group, with other groups including students with BMI 85–94% (overweight category) and students with BMI ≥ 95% (obese category) [37].

### 2.3. Data Analysis

Summary statistics were used to quantify sample characteristics, including FI, physical activity, BMI, sleep duration, food and drink patterns, housing instability, and demographics. Tetrachoric correlations were performed to assess relationships between FI, receipt of free or reduced-price lunch, and housing instability; analyses revealed independence of all variables (strongest correlation between FI and housing instability, rho = 0.43). Multivariable logistic regression was conducted to determine if youth-reported past-month FI was significantly associated with dietary behaviors, including meeting minimum levels for fruit and vegetable intake, any milk consumption, SSB consumption, fast food intake, and school-day lunchtime behavior with adjustment for demographic characteristics and indicators for physical and emotional health. In the first model, relationships between FI and food and beverage patterns and behaviors were examined with adjustment for demographic characteristics and housing instability. In a second step, multivariable relationships between FI and food and beverage patterns and behaviors were examined with adjustment for demographic covariates, housing instability and all indicators for youth physical and emotional health.

All analyses were performed using Stata 12 SE (Stata Corp, College Station, TX, USA). Prior to each MSS data release, all surveys revealing a pattern of missing, contradictory, and implausible responses are removed. In the current study, all remaining missing data were posited to be missing at random, and any remaining students with missing data were excluded from analyses.

## 3. Results

### 3.1. Student Demographics

Students’ mean age was 14.8 years (range 12–19 years). Respondents’ biological sex was evenly split between male (49.9%) and female (50.1%) identification. Students’ reported race-ethnic identities included NH American Indian or Alaska Native (1.2%), NH Asian or Asian American (6.6%), NH Black, African or African American (7.8%), NH Native Hawaiian or Other Pacific Islander (0.2%), NH multiple races (8.7%), NH White (69.2%), and Hispanic or Latino/a (6.1%). A majority of students reported no receipt of free or reduced-price lunch (no = 64.7%; yes = 23.6%; don’t know = 11.7%). Nearly equal proportions of participating students resided in the Twin Cities Metro area (53.4%) and Greater Minnesota (46.6%).

### 3.2. Food Insecurity, Housing Instability

Students reported experiences of FI and housing instability. Nearly 1 in 20 students reported experiencing past-month FI (*n* = 5289, 4.5%). Similarly, approximately 5% of respondents (*n* = 5577) reported experiencing past-year housing instability.

### 3.3. Student BMI, Emotional Health and Health-Related Behaviors

Approximately 1 in 4 students had a BMI ≥ 85% (BMI 85–94% [overweight category]: 14.1%; BMI ≥ 95% [obese category]: 11%). Less than half of respondents reported exercising 5–7 days during the past week (46.3%), or sleeping at least 8 h on a typical school night (35.5%). More than 1 in 5 students screened positive for the PHQ-2 (22.4%).

### 3.4. Thresholds for Dietary Behaviors

Few students met minimum levels for past-week fruit and vegetable intake. Less than 1 in 10 students reported consuming vegetables three or more times/day in the past 7 days (8.6%). Approximately 1 in 4 students reported eating fruit two or more times/day in the past week (27.7%). Approximately half of students drank at least one serving of milk/day in the past week (46.4%). Almost 1 in 4 students drank at least one SSB/day over the past week (23.8%); and approximately 1 in 10 students ate fast food four or more times during the past week (11.7%). More than 1 in 10 students reported they usually do not eat lunch (13.1%). Among food-insecure students, almost 1 in 3 reported they usually do not eat lunch (30%).

### 3.5. Past-Month Food Insecurity and Past-Week Dietary Behaviors

Student-reported past-month FI was significantly associated with past-week dietary behaviors (Table 1, Table 2, Table 3, Table 4 and Table 5). Net of covariates including sociodemographics and emotional and physical health indicators, student report of FI was associated with reduced odds of meeting minimum thresholds for daily vegetable (adjusted odds ratio [AOR]: 0.86, 95% confidence interval [CI]: 0.75–0.98), fruit (AOR: 0.76, 95% CI: 0.69–0.82), and milk (AOR: 0.79, 95% CI: 0.73–0.84) intake. Alternately, student report of past-month FI was associated with increased odds of low-quality dietary intake, including daily SSB consumption (AOR: 1.21, 95% CI: 1.12–1.30) and consuming fast food four or more times (AOR: 1.62, 95% CI: 1.49–1.76) in the past week.

### 3.6. Past-Year Housing Instability and Past-Week Dietary Behaviors

In fully adjusted analytic models, student report of past-year housing instability was significantly associated with increased odds of low-quality dietary intake, including daily sugar-sweetened beverage consumption and frequent fast food dining (Table 4 and Table 5).

### 3.7. Emotional and Physical Health Indicators and Dietary Behaviors

Analytic models also revealed significant associations between emotional and physical health indicators and student dietary behavior. Screening positive on the PHQ-2 was associated with reduced odds of meeting minimum thresholds for daily fruit and milk intake, and increased odds of daily SSB consumption and frequent fast food intake (Table 2, Table 3, Table 4 and Table 5).

Student participation in physical activity at least 5 times in the past week was associated with increased odds of meeting minimum thresholds for vegetable, fruit, and milk consumption, as well as increased odds of drinking SSB at least daily (Table 1, Table 2, Table 3 and Table 4).

Student report of getting at least 8 h of sleep on a typical school night was associated with better-quality dietary behaviors, including increased odds of meeting minimum thresholds for daily vegetable, fruit, and milk consumption, and reduced odds of daily SSB intake and frequent fast food dining over the past week (Table 1, Table 2, Table 3, Table 4 and Table 5).

In fully adjusted multivariable models, student BMI ≥ 85% was associated with few dietary measures, including increased odds of daily SSB intake and reduced odds of daily consumption of two or more fruits during the past week (Table 2 and Table 4).

### 3.8. School Lunch Behavior

Past-month FI was associated with student increased odds of not eating lunch during a typical school day (Table 6); relationships are noted even with adjustment for student participation in the National School Lunch Program. Additional covariates were significantly associated with student report of not eating lunch on a typical school day, including student experience of past-year housing instability, positive PHQ-2 screen, and youth BMI ≥ 85%. Physical activity on at least 5 days in the past week and sleeping at least 8 h on a typical school night were associated with reduced odds of students skipping lunch.

In sub-analyses examining correlates of school lunch behavior among food-insecure students (analytic model adjusted for age, race-ethnic identification, sex, region, housing insecurity, and physical and emotional health indicators), compared to students receiving free or reduced-price lunch, student report of not receiving free or reduced-price lunch (AOR: 1.89, 95% CI: 1.61–2.23), or report of not knowing about receiving free or reduced-price lunch (AOR: 1.60, 95% CI: 1.27–2.01) was associated with increased odds of not eating lunch in a typical school week. Among food-insecure students, additional covariates of not eating lunch included female sex (AOR: 1.59, 95% CI: 1.37–1.86), residing in the Twin Cities Metro region (AOR: 1.25, 95% CI: 1.07–1.45), experience of past-year housing insecurity (AOR: 1.28, 95% CI: 1.07–1.54), and screening positive for depression (AOR: 1.65, 95% CI: 1.43–1.92). For each additional year of age, the odds of skipping lunch among food-insecure students increased approximately 8% (AOR: 1.08, 95% CI: 1.02–1.14). Physical activity on at least 5 days in the past week (AOR: 0.84, 95% CI: 0.72–0.98) and sleeping at least 8 h on a typical school night (AOR: 0.58, 95% CI: 0.46–0.72) were associated with reduced odds of food-insecure students skipping lunch.

## 4. Discussion

### 4.1. Summary of Key Study Findings

Using data from adolescents participating in one of the longest-running youth surveys in the US (Minnesota Student Survey, MSS), this study found significant associations between student-reported past-month food insecurity (FI) and the quality of past-week dietary consumption indicating significantly reduced odds of achieving minimum thresholds for vegetable, fruit, and milk intake, and increased odds of daily SSB and frequent fast food intake. Identified diet-related behaviors present significant risk for poor cardiometabolic health if sustained over time. Among students experiencing past-month FI, compared to students who participated in the National School Lunch Program (NSLP), students who did not participate or were uncertain of NSLP participation had significantly increased odds of not eating lunch.

### 4.2. Results in Context

Study findings are consistent with current literature suggesting an association between household FI and youth reduced vegetable and fruit intake [4,13,14,15,16,17], adolescent reduced intake of diary [17] here, represented by milk, and increased odds of youth SSB consumption [15]. Overall, findings linking FI with low-quality diet, including increased consumption of fast food are also congruent with relationships found for food-insecure adults [4,6,8,9].

Diet quality and consumption of nutrient-dense foods decline as youth move through adolescence [38]. Findings from a recent study using data from the National Health and Nutrition Examination Survey (2015–2018) reveal that although approximately 90% of adolescents ages 12–19 years consume any vegetable on a given day, less than two-thirds of adolescents consume any fruit [39]. The present study found less than 10% of MSS middle and high school students achieved intake of a minimum threshold for vegetables, defined by three or more vegetables/day; and less than one-third of students consumed a minimum level for fruits, specified as two or more fruits/day. Alternately, almost one-quarter of youth reported drinking at least one SSB/day and just over 10% of students consumed fast food at least four times in the previous week. Observed national trends in adolescent dietary behaviors and findings from the current study may mean that adolescents experiencing FI are at even greater risk for poor health related to low-quality dietary intake, as foods with high sugar and fat content may be cheaper to purchase, more readily available at convenient food access points, and provide for greater satiety.

Food-insecure youth have a number of risk factors for poor quality and/or quantity of intake, including eating fewer family meals and breakfasts per week [40]. Even in the setting of family meals, previous research suggests that FI is associated with low-quality foods served at the meal table, including more SSB and less fruits and vegetables when compared to food-secure households [41]. Families must navigate the reality of higher costs for nutrient-dense foods, including fruits and vegetables, when compared to energy-dense foods, such as chips, processed and packaged foods [11]. Similarly, current study findings of youth receipt of free or reduced-price lunch (as an economic indicator) and poor dietary intake, including reduced odds of eating at least two fruit servings/day, and increased odds of daily SSB consumption and frequent fast food intake may reflect relative low cost for the latter when compared to whole fruits. The significant relationships observed between housing instability and increased odds of at least daily SSB and frequent fast food intake also are likely related to cost. Low-priced menu items and sale items found in some convenient food access points may facilitate purchase of greater quantities of foods and beverages that are energy-dense, but nutrient-poor.

The current study suggests that FI among adolescents is also associated with not eating school lunch. Low-income youth from food-insecure households are more likely to participate in school meal programs than are low-income youth from food-secure households [42]. Among food-insecure students in the current study, compared to youth participating in the NSLP, students who did not participate or were uncertain of participation had increased odds of not eating lunch. Thus, for food-insecure students, participation in the NSLP was positively associated with gaining access to essential nutrition during the school day [42]. Indeed, research supports school meal programs in the US as a source for improved diet quality and school performance among youth from low-income and food-insecure households [42]. However, findings from the current study also suggest that among the full sample of youth, participation in the NSLP was associated with student increased odds of not eating lunch during a typical school week. Additional qualitative study may elucidate reasons behind such a finding, including perceived stigma related to participation in supplemental nutrition programs [43,44]. Qualitative study may also be informative for understanding youth and family participation in other nutrition programming. In addition to the NSLP (the largest of the child nutrition programs in the US), the US Department of Agriculture administers the School Breakfast Program, the Summer Food Service Program, and the Child and Adult Care Food Program.

Relationships between FI and adolescent diet-related behaviors were robust to a number of hypothesized student physical and emotional health indicators. Physical and emotional health indicators were also significantly associated with dietary behaviors, generally in expected directions. The measure for vegetable intake does not distinguish between starchy and non-starchy vegetables, and includes potatoes which may account for the positive association between screening positive for the PHQ-2 and vegetable intake in this study. Inclusion of potatoes in the vegetable question may also account for the attenuated strength of the negative association between FI and vegetable intake as compared to FI and fruit intake. The association of physical activity with increased odds of daily SSB consumption may reflect increased availability of sports drinks during sports and team-related activities. The consistency in relationships between sleep and all diet-related behaviors examined in this study is notable. In the current study, sleep was positively related to vegetable, fruit, and milk consumption, and negatively related to SSB and fast food intake. Further, student report of sleeping at least 8 h on a typical school night was associated with reduced odds of skipping lunch. Current findings are consistent with emerging literature identifying significant associations between sleep and diet-related outcomes. For example, in a national cross-sectional study of youth in China, Cao and colleagues found short sleep duration (less than 7 h) to be associated with reduced odds of eating vegetables and fruits among adolescents [45]. Among adults, short sleep duration (generally ≤6 h) has been linked to higher daily caloric intake (not offset by energy expenditures) and lower diet quality [46]. Proposed mechanisms for links between sleep duration and diet-related behavior and quality include alterations in appetite-related hormones, altered sensitivity-connectivity in brain reward centers, and irregularities in mealtimes, such as nighttime eating associated with intake of foods high in fat and sugar content [46].

### 4.3. Clinical, Public Health and Advocacy Implications

Beyond declines in dietary quality known to occur among adolescents, FI during adolescence may portend increased risk for diet-related morbidity, particularly for youth experiencing sustained periods of disruption in access to nutrient-dense foods. Additional study examining longitudinal variation in adolescent food quality and frequency of missed meals in settings of resource limitation may inform needed steps to support youth and families in clinical settings. At the individual level, having conversations with adolescents about food availability and incorporating healthy standards when making decisions about what to eat when on their own is important. As access to health services is integral to school attendance and participation in organized sports (for example in the US), creating care models designed to address adolescents’ increasing autonomy in food and beverage choice is particularly relevant. Connecting anticipatory guidance about food to perceptions of current and future well-being may also support youth healthy decision making.

Innovative partnerships between food resource organizations and schools and/or health services settings may facilitate access to foods, as well as facilitate more nuanced conversations between providers, youth and families to prioritize nutrient density whenever possible, while also allowing for economy of food purchases. For example, integrative nutrition and health programs, in which food distribution programs form community partnerships, using schools as community hubs, to provide nutrition education, health screenings, and opportunities for physical activity represent innovations in food safety net programming [47]. In these programs, food is distributed in a ratio of 3:1, produce to shelf-stable goods [47]. In addition, intervention research should assess the feasibility of clinical care models in which a team of advocates (e.g., nutrition specialist, social work, and community health worker) is accessible to strategize with families as they work to maximize food resources within their control. This may be particularly challenging as usual sources of food are likely inconsistent among individuals and families experiencing FI. However, programs where food pantries are located within clinics or co-located with health services may provide a prototype for such clinical care models [48,49].

There is also a need for optimizing food safety net programming such that all facilities are fully equipped to store fresh fruits and vegetables, and to make these options readily available. Although community food banks and pantries are a vital safety net for adults and youth experiencing FI [50], they have limited capacity to support healthy diets and there is a need to expand capacity to accommodate increased quantities of perishable goods [51,52,53]. Advocacy for policies to standardize manufacturer donations and to support food rating systems that improve the quality of food contributions are needed [52,54]. Policies to support partnerships between pantries and farmers’ markers or farming cooperatives could support stable availability of fresh produce [52]. Recent research among adults has shown client food bag quality, represented by scores for vegetables, greens and fruits, to be correlated with client diet nutrient density scores [55]. Interventions to address food quality in food banks and pantries would support nutrition and healthy eating objectives outlined in Healthy People 2030 [56] for youth and families who rely on such organizations to mitigate the negative impact of disruptions in access to food. The Healthy People Initiative is particularly relevant as it provides measurable 10 year objectives for improving health and well-being of individuals, families, communities, and populations across the US. Healthy People 2030 represents the 5th and current iteration of the Initiative. As in past iterations, objectives for 2030 build on nationwide progress in achieving public health priorities such as reducing chronic disease development and improving preventive behaviors; in addition, objectives for 2030 provide guidance for increased address of health equity and social drivers of health.

### 4.4. Study Limitations

This study has limitations. Data are cross-sectional, and causality and directionality for relationships cannot be established. Respondents for the current study, 8th, 9th, and 11th graders in Minnesota, may not be representative of adolescents attending school in other states and regions of the US and internationally. Further, respondents may not be representative of youth not attending school, or eligible Minnesota youth who did not participate in the online survey Thus, relationships between FI and diet-related behaviors identified in this study are not generalizable to all adolescents.

Although analytic models examining relationships between FI and dietary behaviors included account of confounders for food and beverage behaviors, residual confounding from unmeasured factors or imperfect measurement cannot be ruled out. Within the confines of a survey designed to address broad trends in youth behavior and well-being, the MSS does not include information on youth attitudes about food and nutrition, such that the current analyses cannot disentangle relationships between intake measures and youth overall food preferences. Likewise, as there is no information on the chronicity of adolescents’ experiences of FI, analyses cannot account for early impacts of FI on food preferences later in adolescence (for youth who have been chronically food insecure). Food and beverage questions do not represent an exhaustive menu for intake; however, the range of intake measures available (for middle and high school youth), including fast food and the number of different types of SSB, exceed what is available in other national youth surveys [57]. Alternately, the inclusion of starchy varieties in the single question about vegetables likely limits the ability to discern true relationships between FI and access to green leafy and low carb vegetables [57]. Food insecurity is a complex construct [30,58,59], and the current survey uses a single item to assess youth FI; however, the question is adapted from the USDA food security module, tailored for youth respondents [28,29]. Relationships reference past 7 days of food and beverage consumption, and may not represent usual intake. However, the past 7-day reference is time-congruent with student report of past-month FI.

## 5. Conclusions

Using data from a statewide school-based survey of adolescents in the 8th, 9th, and 11th grades in the United States, this study found significant relationships between youth report of past-month FI and past-week reduced odds of meeting minimum levels for vegetable, fruit, and milk intake. Alternately, FI was associated with SSB and fast food frequencies that portend significant risk for current and future cardiometabolic health if sustained. Study findings suggest the need for more nuanced anticipatory guidance strategies and advocacy actions such that youth and families are supported in prioritizing and accessing nutrient-rich foods without compromising satiety and cost savings.

## Figures and Tables

**Table 1:** Past-Month Food Insecurity and Student Consumption of Vegetables Three or More Times/Day, Past 7 Days.

	Model 1 AOR (95% CI) ^a^	Model 2 AOR (95% CI) ^b^
Past-month food insecurity, yes	0.83 (0.74–0.93) **	0.86 (0.75–0.98) *
**Sociodemographic Indicators**		
Age	0.93 (0.91–0.95) ***	0.97 (0.96–0.99) **
*Biological Sex*		
Biological sex, female	1.18 (1.13–1.23) ***	1.38 (1.32–1.45) ***
*Race-Ethnic Identification*		
NH White	Referent	Referent
NH American Indian or Alaska Native	1.17 (0.96–1.43)	1.24 (0.99–1.56)
NH Asian, Asian American	1.46 (1.34–1.58) ***	1.69 (1.55–1.85) ***
NH Black, African, African American	1.28 (1.17–1.40) ***	1.31 (1.18–1.45) ***
Hispanic, Latino/a	1.29 (1.18–1.42) ***	1.46 (1.31–1.62) ***
NH Native Hawaiian, Pacific Islander	1.58 (1.03–2.43) *	1.77 (1.11–2.81) *
NH multiple races	1.13 (1.04–1.22) **	1.23 (1.13–1.34) ***
*Participation in National School Lunch Program*		
Receipt of free or reduced-price lunch, no	Referent	Referent
Receipt of free or reduced-price lunch, yes	0.88 (0.83–0.93) ***	0.94 (0.88–1.00)
Receipt of free or reduced-price lunch, don’t know	0.93 (0.87–1.00)	0.94 (0.87–1.02)
*Region*		
7-county Twin Cities metro area, yes	1.16 (1.11–1.21) ***	1.23 (1.17–1.29) ***
*Housing*		
Past 12 months housing insecurity, yes	1.13 (1.03–1.25) *	1.10 (0.98–1.23)
**Emotional and Physical Health Indicators**		
Screen positive PHQ-2, yes		1.10 (1.03–1.16) **
Physical activity 5–7 days for at least 60 min in last 7 days, yes		2.12 (2.02–2.23) ***
Sleep duration at least 8 h for typical school night, yes		1.45 (1.38–1.52) ***
BMI < 85%		Referent
BMI 85–94%		0.97 (0.91–1.04)
BMI ≥ 95%		0.92 (0.85–1.00)

Abbreviation: AOR, adjusted odds ratio; CI, confidence interval; NH, Non-Hispanic; PHQ, Patient Health Questionnaire; BMI, body mass index. ^a^ Model 1: odds ratios adjusted for sociodemographic indicators, namely age, biological sex, race-ethnic identification, receipt of free or reduced-price lunch, area-region of residence, and housing insecurity. ^b^ Model 2: odds ratios adjusted for sociodemographic indicators (above), and emotional and physical health indicators. *** *p* < 0.001, ** *p* < 0.01, * *p* < 0.05.

**Table 2:** Past-Month Food Insecurity and Student Consumption of Fruit Two or More Times/Day, Past 7 Days.

	Model 1 AOR (95% CI) ^a^	Model 2 AOR (95% CI) ^b^
Past-month food insecurity, yes	0.68 (0.63–0.74) ***	0.76 (0.69–0.82) ***
**Sociodemographic Indicators**		
Age	0.93 (0.92–0.94) ***	0.97 (0.96–0.98) ***
*Biological Sex*		
Biological sex, female	1.18 (1.15–1.21) ***	1.35 (1.31–1.39) ***
*Race-Ethnic Identification*		
NH White	Referent	Referent
NH American Indian or Alaska Native	1.00 (0.87–1.14)	1.01 (0.87–1.18)
NH Asian, Asian American	0.98 (0.92–1.03)	1.08 (1.02–1.15) *
NH Black, African, African American	1.02 (0.96–1.09)	1.06 (1.00–1.14)
Hispanic, Latino/a	1.11 (1.05–1.18) **	1.23 (1.15–1.32) ***
NH Native Hawaiian, Pacific Islander	1.26 (0.93–1.70)	1.38 (0.99–1.91)
NH multiple races	0.97 (0.92–1.02)	1.06 (1.00–1.11)
*Participation in National School Lunch Program*		
Receipt of free or reduced-price lunch, no	Referent	Referent
Receipt of free or reduced-price lunch, yes	0.71 (0.68–0.74) ***	0.76 (0.73–0.80) ***
Receipt of free or reduced-price lunch, don’t know	0.74 (0.71–0.77) ***	0.77 (0.74–0.81) ***
*Region*		
7-county Twin Cities metro area, yes	1.36 (1.32–1.40) ***	1.43 (1.39–1.47) ***
*Housing*		
Past 12 months housing insecurity, yes	0.98 (0.91–1.04)	0.99 (0.92–1.06)
**Emotional and Physical Health Indicators**		
Screen positive PHQ-2, yes		0.87 (0.84–0.91) ***
Physical activity 5–7 days for at least 60 min in last 7 days, yes		1.91 (1.86–1.97) ***
Sleep duration at least 8 h for typical school night, yes		1.37 (1.32–1.41) ***
BMI < 85%		Referent
BMI 85–94%		0.91 (0.87–0.95) ***
BMI ≥ 95%		0.85 (0.81–0.90) ***

Abbreviation: AOR, adjusted odds ratio; CI, confidence interval; NH, Non-Hispanic; PHQ, Patient Health Questionnaire; BMI, body mass index. ^a^ Model 1: odds ratios adjusted for sociodemographic indicators, namely age, biological sex, race-ethnic identification, receipt of free or reduced-price lunch, area-region of residence, and housing insecurity. ^b^ Model 2: odds ratios adjusted for sociodemographic indicators (above), and emotional and physical health indicators. *** *p* < 0.001, ** *p* < 0.01, * *p* < 0.05.

**Table 3:** Past-Month Food Insecurity and Student Consumption of Milk One or More Times/Day, Past 7 Days.

	Model 1 AOR (95% CI) ^a^	Model 2 AOR (95% CI) ^b^
Past-month food insecurity, yes	0.71 (0.67–0.76) ***	0.79 (0.73–0.84) ***
**Sociodemographic Indicators**		
Age	0.90 (0.89–0.91) ***	0.93 (0.92–0.94) ***
*Biological Sex*		
Biological sex, female	0.44 (0.43–0.45) ***	0.47 (0.46–0.48) ***
*Race-Ethnic Identification*		
NH White	Referent	Referent
NH American Indian or Alaska Native	0.64 (0.57–0.72) ***	0.67 (0.58–0.76) ***
NH Asian, Asian American	0.72 (0.68–0.76) ***	0.78 (0.73–0.82) ***
NH Black, African, African American	0.72 (0.68–0.76) ***	0.72 (0.67–0.76) ***
Hispanic, Latino/a	0.60 (0.57–0.64) ***	0.63 (0.59–0.67) ***
NH Native Hawaiian, Pacific Islander	0.70 (0.53–0.94) *	0.69 (0.50–0.94) *
NH multiple races	0.69 (0.66–0.72) ***	0.73 (0.69–0.76) ***
*Participation in National School Lunch Program*		
Receipt of free or reduced-price lunch, no	Referent	Referent
Receipt of free or reduced-price lunch, yes	0.90 (0.87–0.93) ***	0.95 (0.91–0.98) **
Receipt of free or reduced-price lunch, don’t know	0.85 (0.82–0.89) ***	0.89 (0.85–0.93) ***
*Region*		
7-county Twin Cities metro area, yes	0.82 (0.80–0.84) ***	0.83 (0.81–0.86) ***
*Housing*		
Past 12 months housing insecurity, yes	0.95 (0.90–1.01)	0.97 (0.91–1.04)
**Emotional and Physical Health Indicators**		
Screen positive PHQ-2, yes		0.86 (0.83–0.89) ***
Physical activity 5–7 days for at least 60 min in last 7 days, yes		1.46 (1.42–1.50) ***
Sleep duration at least 8 h for typical school night, yes		1.40 (1.36–1.44) ***
BMI < 85%		Referent
BMI 85–94%		0.97 (0.93–1.00)
BMI ≥ 95%		1.02 (0.98–1.07)

Abbreviation: AOR, adjusted odds ratio; CI, confidence interval; NH, Non-Hispanic; PHQ, Patient Health Questionnaire; BMI, body mass index. ^a^ Model 1: odds ratios adjusted for sociodemographic indicators, namely age, biological sex, race-ethnic identification, receipt of free or reduced-price lunch, area-region of residence, and housing insecurity. ^b^ Model 2: odds ratios adjusted for sociodemographic indicators (above), and emotional and physical health indicators. *** *p* < 0.001, ** *p* < 0.01, * *p* < 0.05.

**Table 4:** Past-Month Food Insecurity and Student Consumption of Sugar-Sweetened Beverage One or More Times/Day, Past 7 Days.

	Model 1 AOR (95% CI) ^a^	Model 2 AOR (95% CI) ^b^
Past-month food insecurity, yes	1.36 (1.28–1.46) ***	1.21 (1.12–1.30) ***
**Sociodemographic Indicators**		
Age	1.08 (1.07–1.10) ***	1.07 (1.06–1.08) ***
*Biological Sex*		
Biological sex, female	0.66 (0.64–0.68) ***	0.63 (0.61–0.65) ***
*Race-Ethnic Identification*		
NH White	Referent	Referent
NH American Indian or Alaska Native	1.47 (1.31–1.66) ***	1.41 (1.24–1.61) ***
NH Asian, Asian American	0.72 (0.67–0.77) ***	0.72 (0.67–0.78) ***
NH Black, African, African American	1.33 (1.25–1.41) ***	1.31 (1.23–1.40) ***
Hispanic, Latino/a	1.03 (0.97–1.10)	0.98 (0.92–1.05)
NH Native Hawaiian, Pacific Islander	1.70 (1.28–2.25) ***	1.70 (1.25–2.32) **
NH multiple races	1.11 (1.06–1.17) ***	1.07 (1.01–1.13) *
*Participation in National School Lunch Program*		
Receipt of free or reduced-price lunch, no	Referent	Referent
Receipt of free or reduced-price lunch, yes	1.31 (1.27–1.36) ***	1.26 (1.21–1.31) ***
Receipt of free or reduced-price lunch, don’t know	1.19 (1.14–1.25) ***	1.16 (1.10–1.22) ***
*Region*		
7-county Twin Cities metro area, yes	0.79 (0.76–0.81) ***	0.78 (0.76–0.81) ***
*Housing*		
Past 12 months housing insecurity, yes	1.63 (1.53–1.72) ***	1.52 (1.42–1.62) ***
**Emotional and Physical Health Indicators**		
Screen positive PHQ-2, yes		1.45 (1.40–1.50) ***
Physical activity 5–7 days for at least 60 min in last 7 days, yes		1.10 (1.07–1.14) ***
Sleep duration at least 8 h for typical school night, yes		0.77 (0.75–0.80) ***
BMI < 85%		Referent
BMI 85–94%		1.05 (1.01–1.10) *
BMI ≥ 95%		1.14 (1.09–1.20) ***

Abbreviation: AOR, adjusted odds ratio; CI, confidence interval; NH, Non-Hispanic; PHQ, Patient Health Questionnaire; BMI, body mass index. ^a^ Model 1: odds ratios adjusted for sociodemographic indicators, namely age, biological sex, race-ethnic identification, receipt of free or reduced-price lunch, area-region of residence, and housing insecurity. ^b^ Model 2: odds ratios adjusted for sociodemographic indicators (above), and emotional and physical health indicators. *** *p* < 0.001, ** *p* < 0.01, * *p* < 0.05.

**Table 5:** Past-Month Food Insecurity and Student Consumption of Fast Food Four or More Times, Past 7 Days.

	Model 1 AOR (95% CI) ^a^	Model 2 AOR (95% CI) ^b^
Past-month food insecurity, yes	1.81 (1.68–1.95) ***	1.62 (1.49–1.76) ***
**Sociodemographic Indicators**		
Age	1.16 (1.15–1.18) ***	1.15 (1.13–1.17) ***
*Biological Sex*		
Biological sex, female	0.86 (0.82–0.89) ***	0.84 (0.80–0.87) ***
*Race-Ethnic Identification*		
NH White	Referent	Referent
NH American Indian or Alaska Native	1.49 (1.28–1.74) ***	1.54 (1.30–1.83) ***
NH Asian, Asian American	1.20 (1.11–1.30) ***	1.22 (1.12–1.33) ***
NH Black, African, African American	2.45 (2.29–2.62) ***	2.42 (2.25–2.61) ***
Hispanic, Latino/a	1.75 (1.63–1.88) ***	1.72 (1.59–1.87) ***
NH Native Hawaiian, Pacific Islander	2.34 (1.66–3.28) ***	2.20 (1.51–3.20) ***
NH multiple races	1.43 (1.34–1.53) ***	1.37 (1.28–1.47) ***
*Participation in National School Lunch Program*		
Receipt of free or reduced-price lunch, no	Referent	Referent
Receipt of free or reduced-price lunch, yes	1.50 (1.43–1.57) ***	1.46 (1.38–1.53) ***
Receipt of free or reduced-price lunch, don’t know	1.36 (1.28–1.45) ***	1.29 (1.20–1.28) ***
*Region*		
7-county Twin Cities metro area, yes	1.03 (0.99–1.08)	1.04 (0.99–1.08)
*Housing*		
Past 12 months housing insecurity, yes	1.80 (1.67–1.93) ***	1.58 (1.45–1.71) ***
**Emotional and Physical Health Indicators**		
Screen positive PHQ-2, yes		1.45 (1.38–1.52) ***
Physical activity 5–7 days for at least 60 min in last 7 days, yes		0.97 (0.93–1.01)
Sleep duration at least 8 h for typical school night, yes		0.81 (0.77–0.85) ***
BMI < 85%		Referent
BMI 85–94%		0.97 (0.92–1.03)
BMI ≥ 95%		0.97 (0.90–1.03)

Abbreviation: AOR, adjusted odds ratio; CI, confidence interval; NH, Non-Hispanic; PHQ, Patient Health Questionnaire; BMI, body mass index. ^a^ Model 1: odds ratios adjusted for sociodemographic indicators, namely age, biological sex, race-ethnic identification, receipt of free or reduced-price lunch, area-region of residence, and housing insecurity. ^b^ Model 2: odds ratios adjusted for sociodemographic indicators (above), and emotional and physical health indicators. *** *p* < 0.001, ** *p* < 0.01, * *p* < 0.05.

**Table 6:** Past-Month Food Insecurity and Student Odds of Not Eating Lunch.

	Model 1 AOR (95% CI) ^a^	Model 2 AOR (95% CI) ^b^
Past-month food insecurity, yes	2.43 (2.27–2.61) ***	1.80 (1.67–1.95) ***
**Sociodemographic Indicators**		
Age	1.10 (1.08–1.11) ***	1.04 (1.02–1.05) ***
*Biological Sex*		
Biological sex, female	1.89 (1.82–1.96) ***	1.63 (1.56–1.70) ***
*Race-Ethnic Identification*		
NH White	Referent	Referent
NH American Indian or Alaska Native	1.48 (1.27–1.72) ***	1.46 (1.23–1.72) ***
NH Asian, Asian American	1.15 (1.07–1.24) ***	1.13 (1.04–1.22) **
NH Black, African, African American	1.36 (1.26–1.46) ***	1.40 (1.29–1.52) ***
Hispanic, Latino/a	1.74 (1.62–1.86) ***	1.63 (1.50–1.77) ***
NH Native Hawaiian, Pacific Islander	2.11 (1.50–2.95) ***	2.12 (1.46–3.07) ***
NH multiple races	1.71 (1.61–1.81) ***	1.50 (1.41–1.61) ***
*Participation in National School Lunch Program*		
Receipt of free or reduced-price lunch, no	Referent	Referent
Receipt of free or reduced-price lunch, yes	1.19 (1.14–1.25) ***	1.09 (1.03–1.14) **
Receipt of free or reduced-price lunch, don’t know	1.44 (1.36–1.52) ***	1.41 (1.32–1.50) ***
*Region*		
7-county Twin Cities metro area, yes	1.02 (0.99–1.06)	1.02 (0.98–1.06)
*Housing*		
Past 12 months housing insecurity, yes	1.40 (1.30–1.51) ***	1.30 (1.19–1.41) ***
**Emotional and Physical Health Indicators**		
Screen positive PHQ-2, yes		2.39 (2.29–2.49) ***
Physical activity 5–7 days for at least 60 min in last 7 days, yes		0.80 (0.77–0.83) ***
Sleep duration at least 8 h for typical school night, yes		0.49 (0.46–0.51) ***
BMI < 85%		Referent
BMI 85–94%		1.16 (1.10–1.23) ***
BMI ≥ 95%		1.21 (1.14–1.29) ***

Abbreviation: AOR, adjusted odds ratio; CI, confidence interval; NH, Non-Hispanic; PHQ, Patient Health Questionnaire; BMI, body mass index. ^a^ Model 1: odds ratios adjusted for sociodemographic indicators, namely age, biological sex, race-ethnic identification, receipt of free or reduced-price lunch, area-region of residence, and housing insecurity. ^b^ Model 2: odds ratios adjusted for sociodemographic indicators (above), and emotional and physical health indicators. *** *p* < 0.001, ** *p* < 0.01, * *p* < 0.05.

## Data Availability

Restrictions apply to the availability of the Minnesota Student Survey data. Data were obtained from the Minnesota Department of Health, Center for Health Statistics. Please visit https://www.health.state.mn.us/data/mchs/surveys/mss/index.html to obtain information about the data user agreement and request form.
